# A rare case report of infective endocarditis caused by *Enterococcus gallinarum* following renal laser lithotripsy

**DOI:** 10.1097/MD.0000000000040802

**Published:** 2024-12-06

**Authors:** Ruoxin Wang, Meng Lv, Liang Fu, Jinlong Zhao, Yinkai Ni, Tianyu Li, Zonghui Chen, Feng Li

**Affiliations:** a Department of Cardiovascular Surgery, The Shanghai Sixth People’s Hospital, Shanghai, China; b Genoxor Medical Science and Technology Inc., Shanghai, China.

**Keywords:** *Enterococcus gallinarum*, infective endocarditis (IE), metagenomic next-generation sequencing, mNGS

## Abstract

**Rationale:**

*Enterococcus gallinarum* is a part of the normal fecal microbiota in the general population and animals, and is rarely isolated in clinical specimens. Due to the increasing use of immunosuppressants, invasive treatments, and overuse of antibiotics, infections caused by enterococci are gradually increasing.

**Patient concerns:**

A 48-year-old man was admitted to our hospital due to a persistent fever for 1 month after renal laser lithotripsy.

**Diagnoses:**

The cardiac ultrasound showed a mass on the mitral valve leaflet of the left atrium. The metagenomic next-generation sequencing test results of blood and vegetation were positive, reporting a large number of characteristic reads of *E. gallinarum*. The patient was diagnosed with infective endocarditis.

**Interventions:**

Mechanical mitral valve replacement was performed, and daptomycin was administered during the perioperative period.

**Outcomes:**

After 4 weeks of antibiotic treatment, the patient’s inflammatory indexes were normal, and no abnormalities such as fever were found. Blood culture and metagenomic next-generation sequencing test results were negative. The patient was then discharged from the hospital.

**Lessons:**

This case emphasizes the possibility of *E. gallinarum* developing severe invasive infections after kidney surgery. Clinical doctors should strengthen their understanding of this type of bacteria, understand their sensitive characteristics and treatment principles.

## 1. Introduction

Infective endocarditis (IE) is a series of infectious diseases characterized by inflammatory manifestations. It is usually caused by microorganisms (bacteria, fungi, viruses, rickettsiae, chlamydia, spirochetes, etc) invading the heart valves or the inner membrane of the ventricular wall. In recent years, the incidence of IE has increased due to the development of cardiac interventional examination and treatment, long-term intravascular catheters, hormones, and immunosuppressive agents, and the increase of intravenous drug users. A Spanish report showed that cardiac device-related IE increased from 11.4% to 27.4% between 1997 and 2004.^[1]^ In the United States, the incidence was 9.3 per 100,000 in 1998 and increased to 15 per 100,000 in 2011.^[2]^ More than 80% of IE^[3]^ are caused by *Staphylococci* (*Staphylococcus aureus* and coagulase-negative Staphylococci), *Streptococci* (*Streptococcus viridans*), and *Enterococci*. The most common species causing infective endocarditis are *Enterococcus faecalis* and *Enterococcus faecium* in *Enterococci*.^[4]^ *Enterococcus gallinarum* (*E. gallinarum*), part of the intestinal flora, is a rare enterococcus that causes endocarditis.^[5]^

Blood culture is the most important method for diagnosing IE. But the negative rate of blood culture is high, up to 20% −60%.^[6]^ Causes of negative results mainly include the use of broad-spectrum antibiotics before blood culture, low pathogen concentration, and difficulty in culturing some bacteria. Some studies have suggested that for patients with surgical indications for infective endocarditis, the survival rate of blood culture results from positive to negative before surgery is much higher than that of patients with continuous blood culture positive.^[7]^ However, the high negative rate of blood culture is difficult to meet the clinical needs for surgical timing judgment. Metagenomic next-generation sequencing (mNGS) is a method that can directly detect all microorganisms in a sample without culture and prediction. In February 2020, mNGS was written as a new early diagnostic method into the expert consensus on bloodstream infection in critically ill patients.^[8]^

## 2. Case presentation

**Figure 1.**
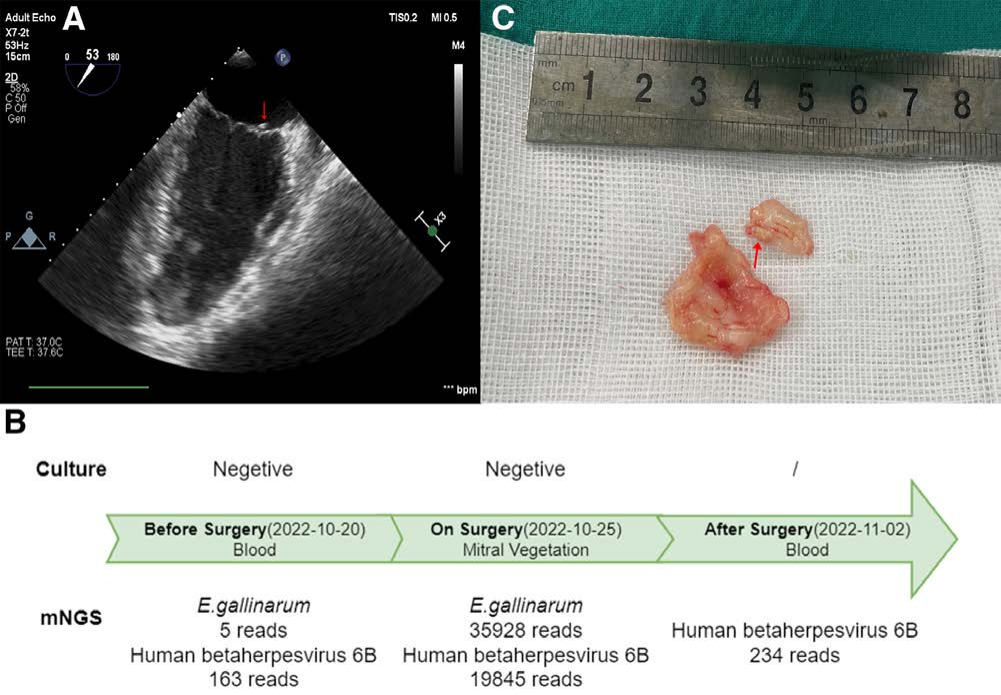
The diagnosis of infection. (A) Cardiac ultrasound. Arrows indicate the observed endocardial vegetations. (B) Pathogenic microorganisms detected by culture mNGS before and on surgery. (C) Heart valve vegetation obtained during surgery. mNGS = metagenomic next-generation sequencing.

A 48-year-old man was admitted to the hospital suffering from a 1-month fever after renal laser lithotripsy. Bacteriological culture was performed on kidney stones obtained from surgery, and the result showed the growth of *E. gallinarum*. He underwent several attempts at empirical antibiotic treatment such as levofloxacin and linezolid, but was no regression and progressive weakness. The patient was diagnosed with an acute myocardial infarction 9 years ago and a permanent stent was implanted in the left anterior descending coronary artery. The following physical findings: body temperature 37.8°C, blood pressure 130/85 mm Hg, pulse rate 80/minute, and mild to a moderate diastolic heart murmur at the apex on auscultation. Blood routine test: white blood cell count of 6.7 × 10^9^/L (normal range, 4–10 × 10^9^/L), neutrophil count 4.6 × 10^9^/L (normal range, 2–7 × 10^9^/L), hemoglobin of 117 g/L (normal range, 120–160 g/L), and platelet count of 199 × 10^9^/L (normal range, 100–300 × 10^9^/L). Other inflammatory markers test: C-reactive protein of 29.85 mg/L (normal range, 0–10 mg/L), erythrocyte sedimentation rate of 42 mm/hour (normal range, 0–20 mm/h), interleukin-1 of 15.6 pg/mL (normal range, 0–5 pg/mL), interleukin-6 of 12.0 pg/mL (normal range, 0–7 pg/mL), tumor necrosis factor-α of 18.4 pg/mL (normal range, 0–4.6 pg/mL), and interferon-α of 8.6 pg/mL (normal range, 0–7.42 pg/mL). Biochemical test: creatinine of 64.9 µmol/L (normal range, 53–106 μmol/L), and uric acid of 418 µmol/L (normal range, 208–428 μmol/L). In addition, the routine urine test showed no abnormal indicators. Cardiac ultrasound showed that there were several hyperechoic masses (3–4 mm) on the mitral leaflets in the left atrium (Fig. 1A). The left atrial anterior-posterior diameter was 44 mm, the left ventricular anterior-posterior diameter (diastolic) was 56 mm, the left ventricular anterior–posterior diameter (systolic) was 39 mm, and the ejection fraction was 47%.

Before surgery the blood culture tests which were drawn in the next 3 continues day showed negative and the blood mNGS showed 5 sequences specific for *E. gallinarum* (Fig. 1B). Daptomycin treatment (500 mg Qd) was started. Mitral mechanical valve replacement was performed under general anesthesia after all. Mitral vegetation was removed surgically as shown in Figure 1C. The mitral vegetation was detected by culture and mNGS. The results of the valve culture were negative, and mNGS were positive (*E. gallinarum*, 35,928 reads, no vancomycin resistance gene; Fig. 1B). Daptomycin treatment (500 mg Qd) was performed perioperative period. After 4 weeks of antibiotic treatment, the patient’s inflammatory indexes were normal, the clinical status was good, the blood culture was negative still, detection of mNGS was negative again, and the drug was stopped (Fig. 1B). No abnormalities such as fever were found after continuous observation.

## 3. Discussion

Our patient had no medical history of cardiac valve disease previously. He only had a history of renal laser lithotripsy and the kidney stone culture harvested from the procedure showed *E. gallinarum*. These may be the source of the pathogen. After a literature search (Medline and Pubmed), infectious endocarditis on native heart valves causing by *E. gallinarum* is extremely rare in immunocompetent patients. Reid et al^[9]^ described the first case of *E. gallinarum* endocarditis in a patient with a bicuspid aortic valve in 2001. The initial source of bacteremia leading to endocarditis is usually the genitourinary or gastrointestinal tract. According to our search, 5 cases of left-sided native valve endocarditis were reported in medical history, and a few infectious endocarditis cases identified the *E. gallinarum* as the pathogenic bacteria in mitral and (or) aortic vegetations in patients with a history of cholecystectomy, gastrectomy, diabetes or immunocompromised hosts.^[10]^

Von Willebrand factor (vWF) is an adhesive glycoprotein that mediates platelet adhesion to damaged endothelium. And in *Staphylococcus*^[11]^ and *Streptococcus pneumonia*^[12]^ infections (such as endocarditis), vWF mediates the interaction between bacteria and vascular endothelium. Liesenborghs et al^[11]^ found that local inflammation and the resulting release of VWF allowed *S. lugdunensis* to bind and colonize heart valves. In vitro, experiments by Claes et al^[13]^ found that adding vWF increased the adhesion ability of wild *Staphylococcus aureus*. Before the surgery we measured vWF: Ag (185.7%, normal 42–140.8%) and vWF: Ac (172.5%, normal 40.3–125.9%) in the peripheral blood of the patient by immunoturbidimetry, which were higher than normal levels. This may be related to *E. gallinarum* adhesion, which may be associated with the development of endocarditis eventually.

*E. gallinarum* can cause infectious endocarditis in immunocompetent humans. Once genitourinary or gastrointestinal tract surgery is performed, it must be paid attention to and treated adequately, and be vigilant to emerging pathogens like *E. gallinarum* in order to prevent endocarditis. Compared with conventional methods, mNGS exhibits better detection performance, which is beneficial to clinical practice.

## Acknowledgments

We thank the efforts and contributions of the reported patients and all the clinical staff in this study.

## Author contributions

**Data curation:** Ruoxin Wang, Liang Fu.

**Methodology:** Jinlong Zhao.

**Visualization:** Yinkai Ni.

**Writing – original draft:** Ruoxin Wang, Meng Lv.

**Writing – review & editing:** Ruoxin Wang, Tianyu Li, Zonghui Chen, Feng Li.
